# Using circulating tumor DNA to monitor myelodysplastic syndromes status

**DOI:** 10.1002/hon.2649

**Published:** 2019-08-11

**Authors:** Pan Zhao, Jiayue Qin, Weiyi Liu, Qianze Zhu, Teng Fan, Haiyan Xiao, Juan Wang, Gang Huang, Hu Xiaomei

**Affiliations:** ^1^ Department of Hematology, Xiyuan Hospital China Academy of Chinese Medical Sciences Beijing China; ^2^ Graduate School China Academy of Chinese Medical Sciences Beijing China; ^3^ Hematology Annoroad Gene Technology Co. Ltd Beijing China; ^4^ Divisions of Pathology and Experimental Hematology and Cancer Biology Cincinnati Children's Hospital Medical Center Cincinnati Ohio

1

Myelodysplastic syndromes (MDS) are a group of heterogeneous myeloid clonal diseases defined by ineffective hematopoiesis, refractory hematopenia, and high‐risk transformation into acute myeloid leukemia (AML). With the development of next‐generation sequencing (NGS) technology, the pathogenesis of MDS has been gradually understood, and as a result, it was discovered that gene mutations played a key role in the occurrence and development of the disease.^1^ Accurate identification of somatic mutations from all variant calls is conducive to the diagnosis, classification, prognosis stratification, and medication guidance of the MDS patients. Currently, detection of gene somatic mutation for MDS patients is mainly based on the bone marrow (BM) tumor DNA (BM‐tDNA). However, BM sample collection is invasive, expensive, difficult to perform, and may be accompanied by surgically related complications. Peripheral blood plasma circulating tumor DNA (PP‐ctDNA), the nucleosome‐bound DNA fragments in plasma, on the other hand, has been widely used in solid cancers and non‐Hodgkin's lymphoma,^2^, ^3^ but up to now, the application of PP‐ctDNA in MDS has been limited.^4^, ^5^

Here, we present a study of gene detection of 26 patients with MDS in our department from July 2017 to July 2018 through an NGS platform with the 127‐gene panel (Tables S1‐S2). The study protocol was approved by the Clinical Research Ethics Committee of Xiyuan Hospital, China Academy of Chinese Medical Sciences (approval no. 2017XLA019‐2). All patients provided written informed consent to participate in the study. In 25 patients with paired sequencing samples, BM‐tDNA showed a good correlation with PP‐ctDNA on the variant allele frequency (VAF) in 6969 matched variant calls (*R* ^2^ = .7295, *P* < .0001) (Figure 1A). The correlations between BM‐tDNA and PP‐ctDNA in both 5615 SNVs and 1354 INDELs were high (*R* ^2^ = .6493, *P* < .0001; *R* ^2^ = .8947, *P* < .0001) (Figure 1B,C), and the correlation of 52 somatic mutations detected in BM‐tDNA and PP‐ctDNA was also significant (*R* ^2^ = .8272, *P* < .0001) (Figures 1D and S1, Table S3). The result of somatic mutations was consistent with the report of Yeh et al (*R* ^2^ = 0.84, *P* < .0001),^4^ and our data demonstrated that the changes detected in PP‐ctDNA in both variant calls and somatic mutations could reflect the mutation spectrum of the BM‐tDNA in MDS patients.

**Figure 1:**
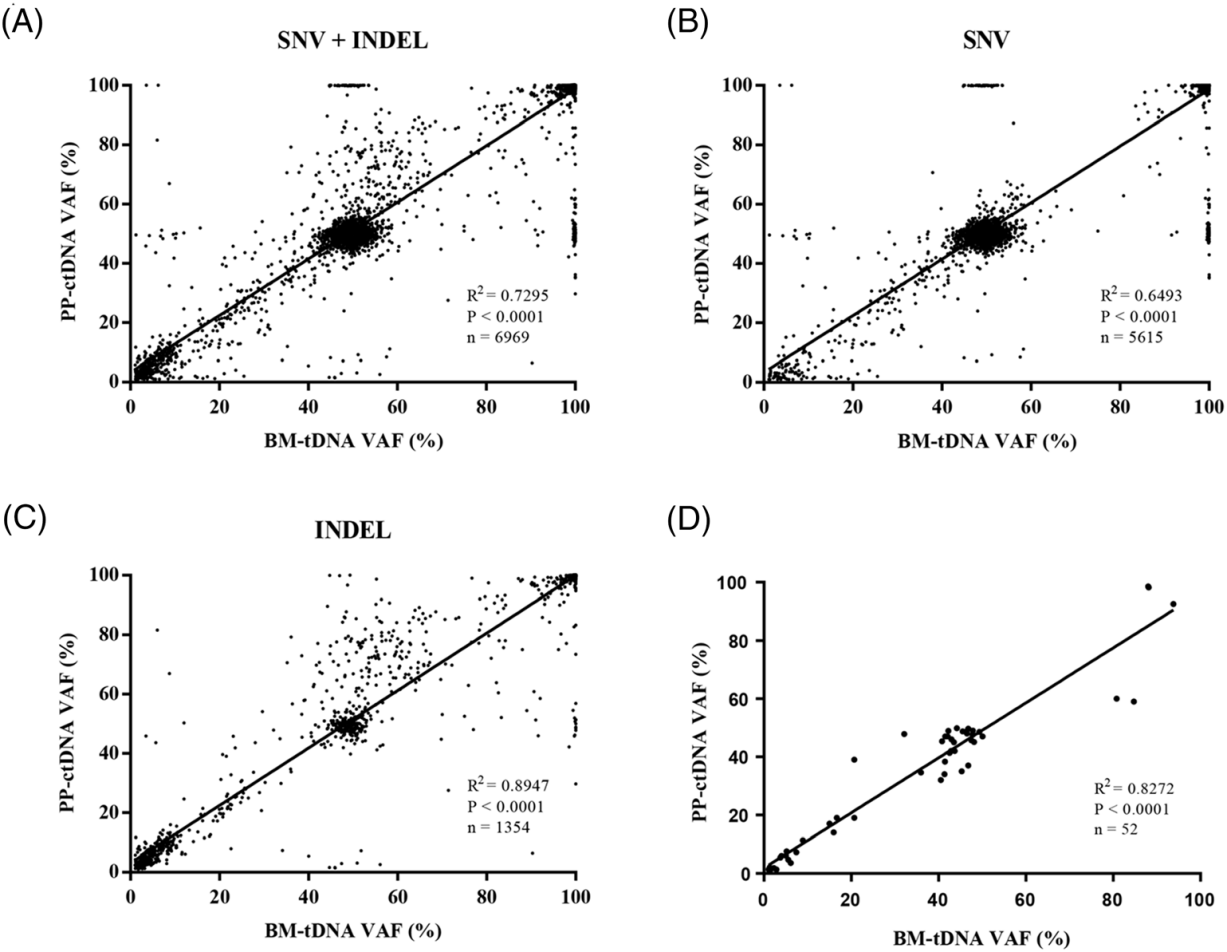
The correlation of VAF in variant calls (A‐C) and somatic mutations (D) between bone marrow tumor DNA (BM‐tDNA) and peripheral blood plasma circulating tumor DNA (PP‐ctDNA) in 25 paired samples of patients. Total variant calls (A) includes SNVs (B) and INDELs (C)

To determine the characteristics of peripheral blood cell tumor DNA (PC‐tDNA), we compared its somatic mutation result with that of BM‐tDNA and PP‐ctDNA in five patients (Figure S2). In Patient 20 (P20) with MDS‐MLD, somatic mutation of *TP53* T231A was detected in BM‐tDNA, PP‐ctDNA, and PC‐tDNA. The respective VAF of this mutation in BM‐tDNA, PP‐ctDNA, and PC‐tDNA was 1.53%, 1.40%, and 1.00% (Figure S2E). The consistency of somatic mutation detection in BM‐tDNA, PP‐ctDNA, and PC‐tDNA was also verified in P1 with MDS‐EB‐1, P6 with MDS‐EB‐2, and P16 with MDS‐EB‐2 (Figure S2A,C,D). However, in P4 with MDS‐SLD, the somatic mutation *BCOR* W1236X was not detected in PC‐tDNA but seen in BM‐tDNA and PP‐ctDNA, and the VAF of the mutation was 6.20% and 3.50%, respectively (Figure S2B). It was speculated that the patient was in the early stage of the disease, so the PC‐tDNA in PB cells could not reflect the mutations in BM‐tDNA. These data suggested that comparing with the PC‐tDNA, the somatic mutations detected in BM‐tDNA could be more comprehensively represented by the PP‐ctDNA, but further testing is needed to validate this observation.

To investigate whether PP‐ctDNA could be used to predict disease status and therapeutic response, serial plasma samples from three patients were used (Figure 2). For P1, who was initially diagnosed with MDS‐EB‐1 and later converted to AML, four somatic mutations *ASXL1* C856fs, *U2AF1* S34Y, *EZH2* A738fs, and *SETBP1* I871T were constantly detected, and two additional somatic mutations of *TET2* K1004fs and *RUNX1* R204Q were found after 15 months (Figure 2A,B). For P3, who was initially diagnosed with MDS‐MLD and later showed SD status after the treatment, the VAF of *TP53* T231A mutation changed from 1.04% to 1.25%, and the VAF of *GNAS* R844C mutation arose from 0% to 1.73% (Figure 2C,D). On the contrary, the VAF of *BCOR* Q1441X decreased from 22.10% to 9.63% in P26 with MDS‐MLD, and the patient later showed a CR status (Figure 2E,F). The PP‐ctDNA monitoring results in three patients showed significant differences in the course of the disease among patients with different outcome, eg, P1 converted to AML, P3 had a SD status, and P26 was evaluated as CR, suggesting the heterogeneity of MDS patients in the diagnosis and treatment process. In P1, two secondary mutations *RUNX1* and *TET2* were detected as the disease progressed. Mutations in *RUNX1* were known to be strongly associated with severe thrombocytopenia (*P* < .001), increased proportion of BM blasts (*P* < .006), and poor overall survival (OS) in MDS patients (hazard ratio [HR], 1.47; *P* < .05).^6^ In contrast, the prognostic significance of *TET2* mutation in MDS patients was still controversial, it was believed to be unrelated to the prognosis or associated with the poor outcomes.^6^, ^7^ However, in AML patients, the significance of *TET2* mutation was relatively clear, and the survival time of patients with the mutation was short.^8^ Therefore, the mutations discovered in these two genes probably correlated with the conversion of MDS to AML. For P3, the *TP53* mutation remained stable for nearly 14 months, and the patient was also in a stable condition; this showed that the *TP53* mutation could be a clear indicator of efficacy, same as described by Welch et al.^9^ Patient P26, on the other hand, reached the CR status over a 13‐month period, and the VAF of *BCOR* Q1441X mutation dropped from 22.10% to 9.63%. It was discovered by Damm et al that patients with *BCOR* mutations may have a significantly inferior OS (HR, 3.3; *P* = .008).^10^ Overall, these data indicated that multiple gene markers in PP‐ctDNA had the dynamic monitoring value to predict disease status and therapeutic response.

**Figure 2:**
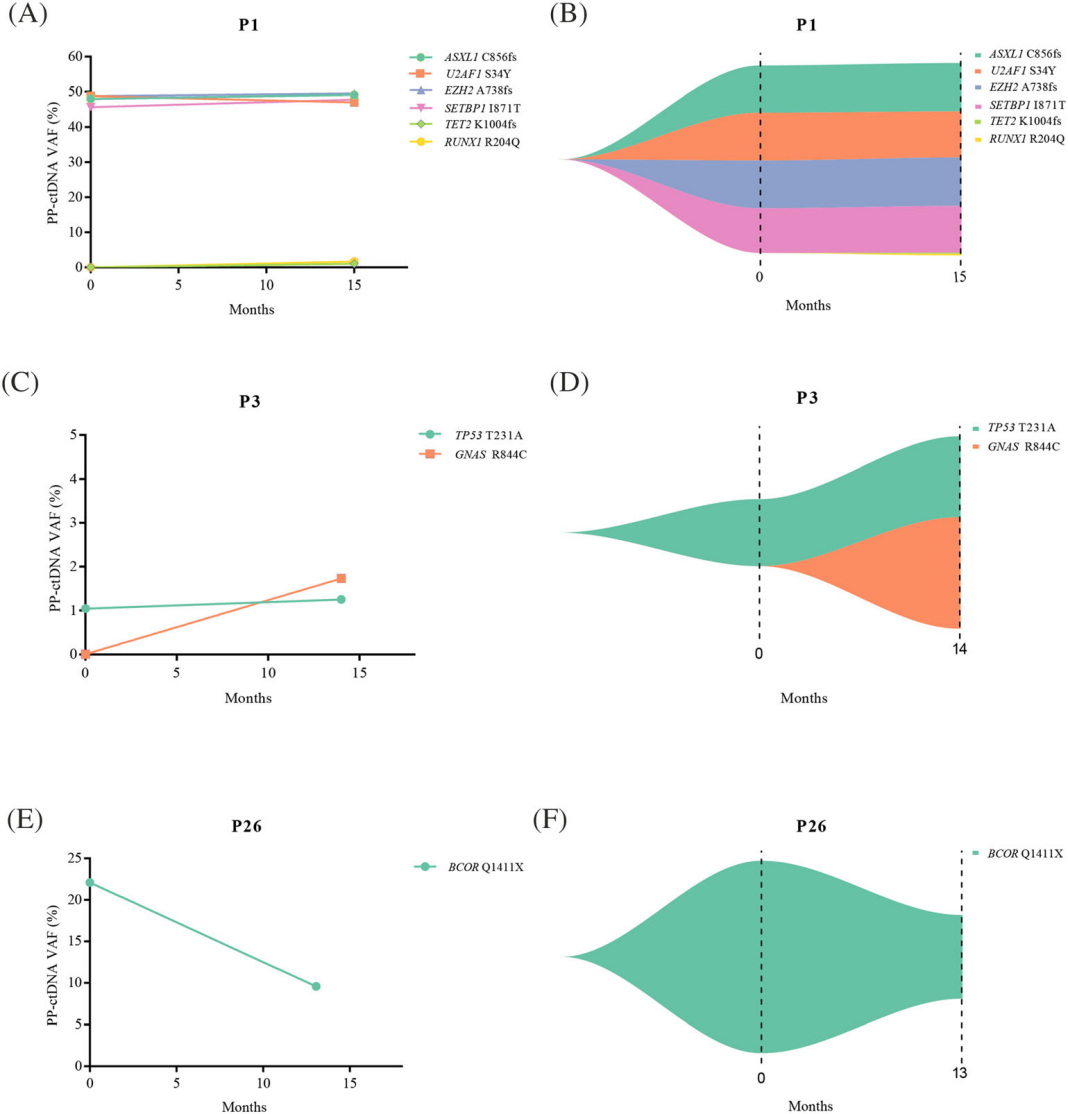
Application of serial peripheral blood plasma circulating tumor DNA (PP‐ctDNA) samples to predict disease status in three patients, including one patient developed AML (A, B), one patient with effect evaluated as SD (C, D), and one patient with effect evaluated as CR (E, F)

In summary, due to the broad‐usage of NGS platform and improving non‐invasive PP‐ctDNA detection technology, our study strongly suggested that the correlations between BM‐tDNA and PP‐ctDNA are high in both variant calls and somatic mutations. To some extent, the abundance of the gene mutations in PP‐ctDNA can predict and reflect disease status and therapeutic response in MDS.

## CONFLICT OF INTEREST

All authors declare that they have no conflict of interests.

## FUNDING INFORMATION

This study was supported by a Grant from the National Natural Science Foundation of China (Nos. 81673821 and 81774142) to Xiaomei Hu and a Grant from the Special Research Foundation of Central Level Public Scientific Research Institutes (ZZ10‐016) to Xiaomei Hu.

## Supporting information

Table S1: Patient information
Table S2: Genetic mutation list in 127‐gene panel
Table S3: Somatic mutations in 25 patients with paired sequencing samples
Figure S1: Mutation heatmap
S2: Comparison of somatic mutations from BM‐tDNA, PP‐ctDNA and PC‐tDNA
