# Supplementary material for: Using circulating tumor DNA to monitor myelodysplastic syndromes status
Source: Hematol Oncol. 2019 Aug 11;37(4):531–3. doi: 10.1002/hon.2649 (PMC6900042; doi:10.1002/hon.2649)
Supplement: Supplementary file 1 — Table S1: Patient information Table S2: Genetic mutation list in 127‐gene panel Table S3: Somatic mutations in 25 patients with paired sequencing samples Figure S1: Mutation heatmap S2: Comparison of somatic mutations from BM‐tDNA, PP‐ctDNA and PC‐tDNA [file HON-37-531-s001.docx]

**Supplementary Methods**

Patients’ characteristics

Karyotype analysis

Detection of variant calls and somatic mutations

Statistical analysis

**Supplementary Tables**

Table S1: Patient information

Table S2: Genetic mutation list in 127-gene panel

Table S3: Somatic mutations in 25 patients with paired sequencing samples

**Supplementary Figures**

Figure S1: Mutation heatmap

Figure S2: Comparison of somatic mutations from BM-tDNA, PP-ctDNA and PC-tDNA

**Supplementary Methods**

**Patients’ characteristics**.

The study population included 26 patients with MDS from the Department of Hematology at Xiyuan Hospital in China, Academy of Chinese Medical Sciences from July, 2017, to July, 2018 (Table S1). The diagnostic criteria, classification criteria, and international prognostic scoring system for MDS were based on the 2007 Vienna standards for the diagnosis of MDS, the 2016 World Health Organization (WHO) classification criteria for MDS, and the 2012 Revised International Prognostic Scoring System (IPSS-R). The ethics committee of Xiyuan Hospital of China Academy of Chinese Medical Sciences approved this research. All subjects provided informed consent in accordance with the Declaration of Helsinki to participate in this clinical trial (registration number: ChiCTR-RNC-17014235).

**Karyotype analysis**.

BM cells were cultured short-term (24 h), G-banding of chromosomes was performed, and karyotypes were determined according to the International Nomenclature System for Human Cytogenetics (ISCN, 2013).

**Detection of variant calls and somatic mutations**.

BM-tDNA, peripheral blood cell tumor DNA (PC-tDNA) and PP-tDNA, were extracted after BM or PB samples were collected from patients. The total amounts of BM-tDNA and PC-tDNA were ≥ 1000 ng with OD_260_/OD_280_ = 1.7-1.9, and total mass of PB-ctDNA is ≥ 20 ng with OD_260_/OD_280_ = 1.7-1.9 for the construction of an Illumina standard library. The Roche NimbleGen liquid phase hybrid capture chip was employed to perform 127-target gene sequencing for high-throughput detection in variant calls and somatic mutations (Supplementary Table S1). The captured exon library was sequenced on the Illumina NextSeq 550AR platform, and each sample in the target area was required to have the average effective depth ≥1000× in BM-tDNA and PC-tDNA, and ≥2000× in PP-ctDNA. By the BWA algorithm to compare the sequence data with the human genome (version: GRCh37), Picard was used to mark the PCR duplication, and the quality value of the sequence alignment results was corrected by means of BaseRecalibrator of GATK. The MuTect2 software was employed for variant detection, and all test results were annotated in the Annovar software. The types of variant call analysis included single nucleotide variants (SNVs) and insertions and deletions (INDELs). SNPs described in human genetic variation databases were excluded for special analysis of somatic mutations. Variant allele frequency (VAF) was calculated as the number of the variant reads divided by the total number of reads for the mutation position. VAF ≥ 1% retained for further analysis. Furthermore, automatic nucleic acid extractor and DNA extraction kit were purchased from RBC Bioscience (Xiamen) Co., Ltd., QPCR kit was purchased from KAPA, and throughput sequencing instrument Nextseq 550AR was purchased from Illumina company.

**Statistical analysis**.

Statistical calculations were performed using Spearman method on GraphPad Prism version 7.00 (GraphPad Software, La Jolla California USA). A P-value of < 0.05 was considered statistically significant.

**Supplementary Tables**

**Table S1. Patient information**

| **Patient** | **Age** | **Sex** | **MDS subtype** | **WBC (/μL)** | **Hb (g/dL)** | **PLT (x1, 000 /μL)** | **ANC (x1, 000 /μL)** | **BM Blasts (%)** | **Karyotype** | **IPSS-R (category)** |
| --- | --- | --- | --- | --- | --- | --- | --- | --- | --- | --- |
| P1 | 31 | M | EB-1 | 3.12 | 72 | 31 | 0.00 | 6.0 | Normal | High |
| P2 | 60 | F | MLD | 5.32 | 67 | 35 | 2.56 | 3.0 | t(1;17) | High |
| P3 | 28 | F | MLD | 4.86 | 85 | 21 | 2.19 | 0.0 | Normal | Low |
| P4 | 39 | F | SLD | 2.65 | 98 | 42 | 1.27 | 1.0 | Complex | High |
| P5 | 77 | M | EB-1 | 3.67 | 88 | 248 | 2.28 | 8.0 | Normal | Intermediate |
| P6 | 69 | M | EB-2 | 12.35 | 75 | 253 | 6.22 | 19 | Normal | High |
| P7 | 47 | M | EB-1 | 2.7 | 122 | 6 | 0.94 | 8.0 | Normal | Intermediate |
| P8 | 69 | F | MLD | 1.90 | 37 | 41 | 1.00 | 1.5 | Normal | Intermediate |
| P9 | 45 | F | SLD | 2.57 | 77 | 54 | 1.33 | 0.0 | 5q- | Intermediate |
| P10 | 81 | M | MLD | 1.91 | 72 | 172 | 1.03 | 1.6 | 20q- | Low |
| P11 | 60 | M | EB-1 | 3.43 | 107 | 42 | 1.02 | 8.0 | Normal | Intermediate |
| P12 | 36 | M | MLD | 4.37 | 54 | 350 | 1.90 | 2.0 | Normal | Low |
| P13 | 25 | F | MLD | 1.9 | 37 | 41 | 1.00 | 1.5 | Normal | Intermediate |
| P14 | 60 | M | EB-1 | 8.33 | 144 | 19 | 5.82 | 6.5 | +8 | High |
| P15 | 43 | F | MLD | 6.48 | 99 | 186 | 2.67 | 3 | Normal | Intermediate |
| P16 | 51 | M | EB-2 | 2.32 | 66 | 16 | 1.16 | 15.0 | Normal | Very High |
| P17 | 29 | F | MLD | 1.1 | 58 | 55 | 0.49 | 5 | Normal | High |
| P18 | 44 | M | MLD | 3.7 | 83 | 24 | 1,60 | 0.0 | Normal | Low |
| P19 | 21 | M | MLD | 3.7 | 112 | 52 | 1.26 | 0 | Normal | Very Low |
| P20 | 20 | M | MLD | 1.9 | 78 | 22 | 0.74 | 0.0 | Normal | Intermediate |
| P21 | 35 | M | RS | 4.61 | 80 | 178 | 1.41 | 0.0 | Normal | Low |
| P22 | 36 | F | MLD | 2.94 | 53 | 23 | 1.5 | 1 | Normal | Intermediate |
| P23 | 45 | F | MLD | 2.6 | 67 | 18 | 0.9 | 1.5 | Normal | Intermediate |
| P24 | 43 | F | MLD | 1.43 | 119 | 103 | 0.68 | 0 | Normal | Very Low |
| P25 | 59 | M | MLD | 2.3 | 73 | 88 | 1.02 | 3 | Normal | Intermediate |
| P26 | 24 | M | MLD | 7.88 | 153 | 113 | 4.32 | 0 | Normal | Very Low |

Abbreviations: M, male; F, female; WBC, white cell blood count; PLT, platelet count; Hb, hemoglobin; ANC, absolute neutrophil count; BM, bone marrow; RS, ring sideroblasts; SLD, single lineage dysplasia, MLD, multilineage dysplasia; EB-1, excess blasts-1; EB-2, excess blasts-2; IPSS-R, revised International Prognostic Scoring System.

**Table S2. Genetic mutation list in 127-gene panel**

| Gene | Transcript | Target Region |
| --- | --- | --- |
| ABCB1 | NM_000927 | Exon 20, 25 |
| ABCC3 | NM_003786 | Exon 1-2, 9, 12-13, 26-27, 29; intron 10, 13 |
| ABL1 | NM_005157 | Exon 4-8 |
| AKT2 | NM_001626 | Exon 2-4, 9-10, 13 |
| AKT3 | NM_181690 | Exon 2, 5 |
| AMER1 | NM_152424 | Exon 1 |
| APC | NM_000038 | Exon 1, 9-10, 15 |
| ASXL1 | NM_015338 | Exon 12 |
| ATM | NM_000051 | Exon 16, 21, 43, 48, 51, 58, 62; intron 36, 40 |
| ATRX | NM_000489 | Exon 8-10, 17-31, 35 |
| BCL2 | NM_000633 | full(1-3) |
| BCOR | NM_001123383 | full(1-14) |
| BCORL1 | NM_021946 | full(1-13) |
| BIRC3 | NM_001165 | Exon 2-9 |
| BLM | NM_000057 | Exon 9-10, 12-20, 37 |
| BRAF | NM_004333 | Exon 1-8, 11-13, 15-18 |
| CACNA1E | NM_001205293 | Exon 4, 5; intron 2 |
| CALR | NM_004343 | Exon 9 |
| CARD11 | NM_032415 | Exon 4-6, 8-9 |
| CBL | NM_005188 | Exon 8-9, 12, 16 |
| CBLB | NM_170662 | Exon 8-10 |
| CBLC | NM_001130852 | Exon 8-10 |
| CCND1 | NM_053056 | Exon 1, 4, 5 |
| CD79B | NM_000626 | Exon 5, 6 |
| CDA | NM_001785 | Exon 1, 2, 4 |
| CDKN2A | NM_000077 | full(1-4) |
| CEBPA | NM_004364 | Exon 1 |
| CREBBP | NM_004380 | Exon 25-28, 31 |
| CRLF2 | NM_022148 | Exon 6 |
| CSF1R | NM_005211 | Exon 6, 21 |
| CSF3R | NM_000760 | Exon 12, 14-17 |
| CTLA4 | NM_005214 | Intron 4 |
| CUX1 | NM_181552 | full(1-24); intron 2, 16, 17, 22, 23 |
| CYP2C19 | NM_000769 | Exon 4, 5 |
| CYP3A4 | NM_017460 | Exon 5, 10 |
| DDX41 | NM_016222 | Exon 5-6, 15 |
| DIS3 | NM_014953 | Exon 10-11, 16 |
| DKC1 | NM_001363 | Exon 1-6, 9-12, 14 |
| DNAH9 | NM_001372 | Intron 52 |
| DNMT3A | NM_022552 | full(1-23) |
| EGFR | NM_005228 | Exon 18-19, 20-21 |
| ELA2 | NM_001972 | Exon 2-5 |
| EP300 | NM_001429 | full |
| ERCC1 | NM_001983 | Exon 4 |
| ERG | NM_001243432 | Exon 2-10 |
| ETNK1 | NM_018638 | Exon 3 |
| ETV6 | NM_001987 | full(1-8) |
| EZH2 | NM_004456 | full(1-20) |
| FAM46C | NM_017709 | Exon 1 |
| FAT1 | NM_005245 | full |
| FBXW7 | NM_033632 | Exon 8-11 |
| FLT3 | NM_004119 | Exon 14-15, 20-21(intron14, intron15) |
| GATA1 | NM_002049 | Exon 2 |
| GATA2 | NM_032638 | Exon 2-6 |
| GATA3 | NM_002051 | Exon 4 |
| GFI1 | NM_005263 | Exon 7 |
| GNAS | NM_080425 | Exon 1, 8, 9 |
| GSTM1 | NM_000561 | full(1-8) |
| GSTP1 | NM_000852 | Exon 5 |
| HAX1 | NM_006118 | Exon 2, 3 |
| HRAS | NM_005343 | Exon 1, 2 |
| ID3 | NM_002167 | Exon 1, 2; intron 1, 2 |
| IDH1 | NM_001282386 | Exon 2, 4 |
| IDH2 | NM_002168 | Exon 4 |
| IKZF1 | NM_006060 | full(1-8) |
| IL7R | NM_002185 | Exon 6 |
| JAK1 | NM_002227 | Exon 9, 12 |
| JAK2 | NM_004972 | exon10, 12, 14, 16 |
| JAK3 | NM_000215 | exon11-13, 15-16 |
| KDM6A | NM_001291415 | full(1-29) |
| KIT | NM_000222 | Exon 2, 8-11, 13-17 |
| KMT2A（MLL） | NM_001197104 | full |
| KMT2C | NM_170606 | Exon 14, 43 |
| KRAS | NM_004985 | Exon 1, 2 |
| MAP2K4 | NM_001281435 | Exon 9 |
| MAP3K7 | NM_145333 | full(1-16) |
| MDM2 | NM_002392 | Exon 3 |
| MEF2B | NM_001145785 | Exon 2-3, 6-8 |
| MLH1 | NM_000249 | Exon 2-3, 13, 15, 19 |
| MPL | NM_005373 | Exon 10 |
| MTHFR | NM_005957 | Exon 4, 7 |
| MYD88 | NM_001172566 | Exon 3-5 |
| NF1 | NM_000267 | full |
| NF2 | NM_000268 | Exon 8, 13 |
| NOTCH1 | NM_017617 | Exon 26, 27, 34 |
| NOTCH2 | NM_024408 | Exon 27, 34 |
| NPM1 | NM_002520 | Exon 10-11, 16 |
| NQO1 | NM_000903 | Exon 5 |
| NRAS | NM_002524 | Exon 1, 2 |
| NT5C2 | NM_012229 | Exon 3-19 |
| NTRK1 | NM_001007792 | Exon 15 |
| NTRK2 | NM_006180 | full(1-21) |
| PDGFRA | NM_006206 | Exon 12, 14, 18 |
| PHF6 | NM_001015877 | full(1-9) |
| PIGA | NM_002641 | full(1-7) |
| PIK3CA | NM_006218 | Exon 9, 20 |
| PRPF8 | NM_006445 | Exon 28 |
| PTEN | NM_000314 | Exon 7, 15 |
| PTPN11 | NM_002834 | Exon 3, 13 |
| RAD21 | NM_006265 | full(1-14） |
| RB1 | NM_000321 | full(1-27) |
| RUNX1 | NM_001754 | full(1-9) |
| SETBP1 | NM_015559 | Exon 3, 4 |
| SETD2 | NM_014159 | Exon 2-5, 8, 12, 14-16, 19-21 |
| SF3B1 | NM_012433 | Exon 13-16 |
| SH2B3 | NM_005475 | Exon 1-3, 6 |
| SMAD4 | NM_005359 | Exon 2,10 |
| SMC1A | NM_006306 | Exon 2-3, 11, 13-14, 16-17 |
| SMC3 | NM_005445 | Exon 9-10, 13, 19, 23, 25, 28 |
| SRP72 | NM_006947 | Exon 6, 7 |
| SRSF2 | NM_003016 | Exon 1, 2 |
| STAG2 | NM_001042749 | full(1-35) |
| STAT3 | NM_003150 | Exon 21 |
| STAT5A | NM_003152 | Exon 15-16, 19, 20 |
| STAT5B | NM_012448 | Exon 14-16 |
| SYK | NM_001135052 | Exon 5 |
| TCF3 | NM_003200 | Exon 17 |
| TERC | NR_001566 | full |
| TERT | NM_198253 | Exon 1-2, 4-12, 14-16 |
| TET2 | NM_001127208 | Exon 1-11 |
| TP53 | NM_000546 | Exon 2-11 |
| TPMT | NM_000367 | Exon 3, 5, 8 |
| TRAF3 | NM_145725 | Exon 1, 3-8, 10 |
| U2AF1 | NM_006758 | Exon 2, 6 |
| WT1 | NM_024426 | Exon 3, 6-9 |
| XRCC1 | NM_006297 | Exon 10 |
| ZRSR2 | NM_005089 | full(1-11) |

**Table S3. Somatic mutations in 25 patients** **with paired sequencing samples**

| **Patient** | **Mutation site** | **VAF in PP-ctDNA** | **VAF in BM-tDNA** |
| --- | --- | --- | --- |
| P1 | NM_015559(SETBP1):c.2612T>C(p.I871T) | 45.68% | 47.50% |
| P1 | NM_006758(U2AF1):c.101C>A(p.S34Y) | 48.81% | 45.50% |
| P1 | NM_015338(ASXL1):c.2568delC(p.C856fs) | 48.00% | 46.50% |
| P1 | NM_004456(EZH2):c.2212dupG(p.A738fs) | 48.75% | 46.40% |
| P2 | NM_015338(ASXL1):c.1852A>T(p.K618X) | 2.00% | 1.30% |
| P2 | NM_001127208(TET2):c.1165_1166insAGGATTCCTTTTCTGCCACT(p.K389fs) | 39.00% | 20.70% |
| P2 | NM_001127208(TET2):c.4639delC(p.Q1547fs) | 47.00% | 50.10% |
| P3 | NM_000546(TP53):c.691A>G(p.T231A) | 1.04% | 1.13% |
| P4 | NM_001123383(BCOR):c.3707G>A(p.W1236X) | 3.50% | 6.20% |
| P5 | NM_002524(NRAS):c.35G>A(p.G12D) | 41.40% | 42.50% |
| P5 | NM_012433(SF3B1):c.1998_1999delinsTG(p.K666_I667delinsNV) | 49.00% | 47.88% |
| P6 | NM_001754(RUNX1):c.496C>T(p.R166X) | 35.00% | 45.33% |
| P6 | NM_012433(SF3B1):c.2098A>G(p.K700E) | 37.00% | 46.78% |
| P6 | NM_000267(NF1):c.6883delT(p.F2295fs) | 59.00% | 84.77% |
| P7 | NM_004364(CEBPA):c.507_508insCTGAA(p.A170fs) | 4.70% | 5.59% |
| P7 | NM_001015877(PHF6):c.346C>T(p.R116X) | 7.30% | 7.41% |
| P7 | NM_002168(IDH2):c.419G>A(p.R140Q) | 14.00% | 15.95% |
| P7 | NM_001282386(IDH1):c.395G>A(p.R132H) | 19.00% | 20.67% |
| P7 | NM_001754(RUNX1):c.1289_1290insTGCC(p.P430fs) | 47.00% | 41.52% |
| P8 | DNMT3A:NM_022552:exon20:c.2360_2370del:p.A787fs | 42.00% | 42.87% |
| P8 | NM_012433(SF3B1):c.2098A>G(p.K700E) | 47.00% | 42.11% |
| P9 | NM_000546(TP53):c.817C>T(p.R273C) | 17.00% | 15.05% |
| P9 | NM_015338(ASXL1):c.1772dupA(p.Y591_Q592delinsX) | 19.00% | 16.72% |
| P11 | NM_006758(U2AF1):c.101C>A(p.S34Y) | 45.00% | 43.50% |
| P14 | NM_015338(ASXL1):c.1888_1910del(p.H630fs) | 34.60% | 35.98% |
| P14 | NM_004456(EZH2):c.1950delT(p.I650fs) | 42.00% | 43.67% |
| P14 | NM_004456(EZH2):c.2069G>A(p.R690H) | 45.10% | 48.16% |
| P14 | NM_001127208(TET2):c.5650A>G(p.T1884A) | 47.60% | 47.81% |
| P14 | NM_005089(ZRSR2):c.44delA(p.H15fs) | 92.60% | 93.80% |
| P16 | ASXL1:NM_015338:exon12:c.G1945T:p.G649X | 32.00% | 40.51% |
| P16 | U2AF1:NM_001025203:exon2:c.C101T:p.S34F | 34.00% | 41.36% |
| P16 | NM_001123383(BCOR):c.4639+1G>A | 60.00% | 80.83% |
| P19 | NM_001123383(BCOR):c.2383A>T(p.K795X) | 38.37% | 41.46% |
| P20 | NM_000546(TP53):c.713G>A(p.C238Y) | 1.40% | 1.53% |
| P21 | NM_021946(BCORL1):c.4773delG(p.M1591fs) | 1.40% | 2.84% |
| P21 | NM_001127208(TET2):c.1249_1273del(p.Q417fs) | 7.60% | 5.18% |
| P21 | NM_012433(SF3B1):c.1873C>T(p.R625C) | 45.40% | 40.81% |
| P22 | NM_001127208(TET2):c.4018C>T(p.L1340F) | 48.64% | 49.35% |
| P23 | NM_015338(ASXL1):c.3938_3947delinsACCCACAA(p.T1313fs) | 3.65% | 6.16% |
| P24 | NM_021946(BCORL1):c.2566C>T(p.Q856X) | 1.90% | 2.23% |
| P24 | NM_005089(ZRSR2):c.55dupA(p.Y18fs) | 5.48% | 3.76% |
| P24 | NM_015338(ASXL1):c.2554_2555insG(p.S852fs) | 46.05% | 42.99% |
| P25 | NM_004985(KRAS):c.35G>A(p.G12D) | 1.41% | 1.08% |
| P25 | NM_002524(NRAS):c.34G>A(p.G12S) | 6.06% | 4.05% |
| P25 | NM_002524(NRAS):c.38G>A(p.G13D) | 6.14% | 5.01% |
| P25 | NM_002524(NRAS):c.35G>A(p.G12D) | 11.36% | 8.91% |
| P25 | NM_003016(SRSF2):c.284_307del(p.95_103del) | 47.88% | 32.13% |
| P25 | NM_004364(CEBPA):c.479_480insAT(p.I160fs) | 48.93% | 42.26% |
| P25 | NM_022552(DNMT3A):c.2207G>A(p.R736H) | 49.71% | 46.79% |
| P25 | NM_080425(GNAS):c.2531G>A(p.R844H) | 49.85% | 44.19% |
| P25 | NM_001042749(STAG2):c.3034C>T(p.R1012X) | 98.21% | 88.16% |
| P25 | NM_001123383(BCOR):c.2264dupA(p.Y755_E756delinsX) | 98.50% | 88.01% |

Abbreviations: VAF, variant allele frequency; PP-ctDNA , peripheral blood plasma circulating tumor DNA; BM-tDNA, bone marrow tumor DNA.

**Supplementary Figures**


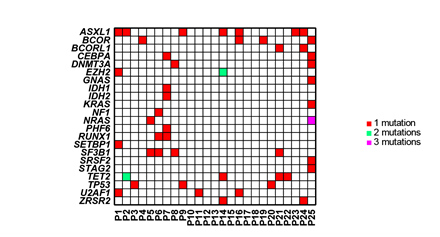
**Figure S1.** Mutation heatmap. Red, green and purple represent one detected mutation, two mutations and three mutations.


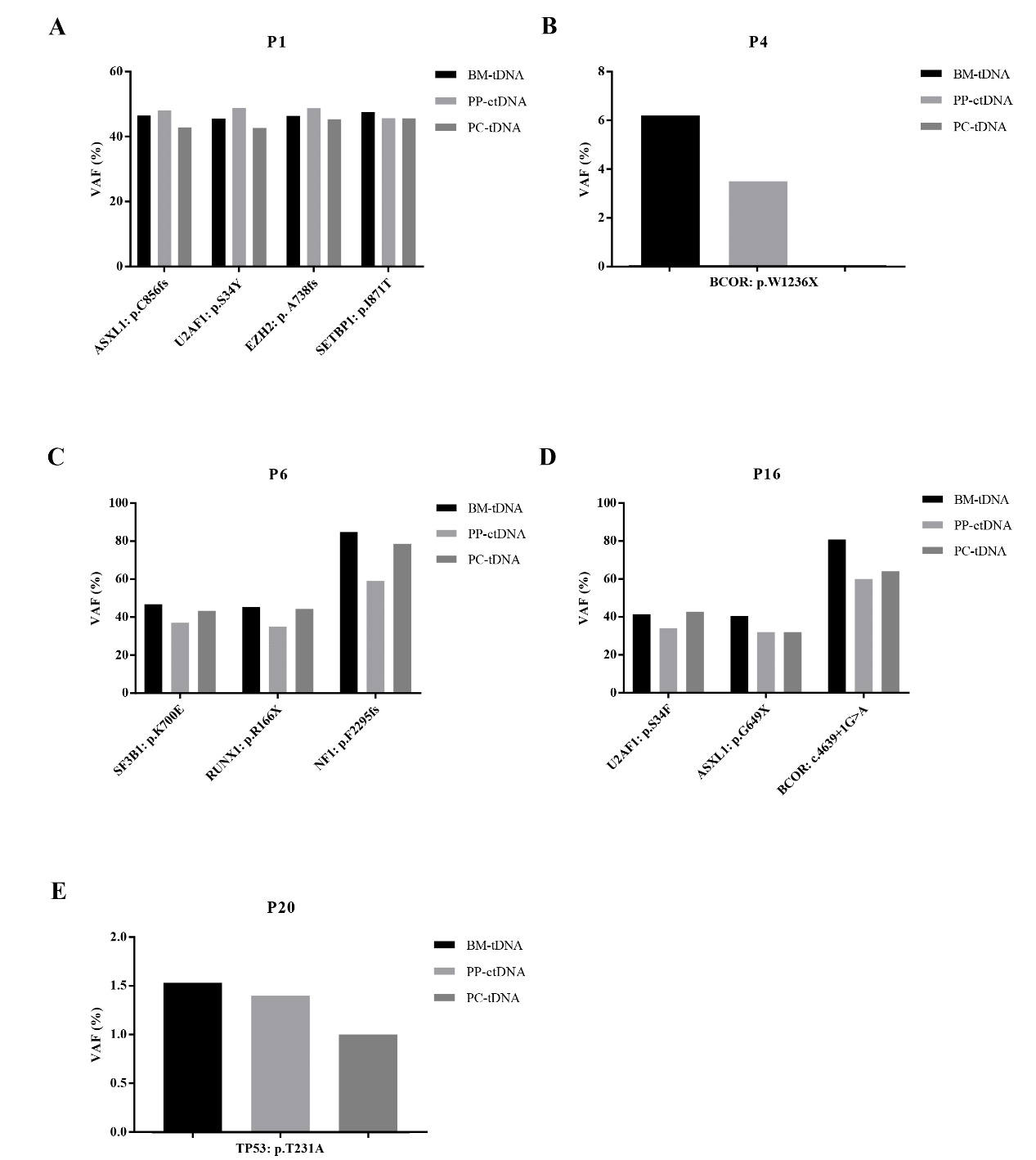


**Figure S2.** Comparison of somatic mutations from bone marrow tumor DNA (BM-tDNA), peripheral blood plasma circulating tumor DNA (PP-ctDNA) and peripheral blood cell tumor DNA (PC-tDNA) in five patients, including one patient with MDS EB-1 (A), one patient with MDS-SLD (B), two patients with MDS-EB-2 (C, D), and one patient with MDS-MLD (E).
